# Supplementary material for: Local norms of cheating and the cultural evolution of crime and punishment: a study of two urban neighborhoods
Source: PeerJ. 2014 Jul 1;2:e450. doi: 10.7717/peerj.450 (PMC4103098; doi:10.7717/peerj.450)
Supplement: Supplemental Information 1 [file peerj-02-450-s001.pdf]

Supplemental Information for:

Local norms of cheating and the cultural evolution of crime and punishment

Kari Britt Schroeder[1,2], Gillian V. Pepper1[1], Daniel Nettle[1]

[1]Centre for Behaviour and Evolution, Newcastle University, Newcastle Upon Tyne, UK

[2]Department of Psychological and Brain Sciences, Boston University, Boston, MA, USA

Supplemental Information consists of one PDF with 15 pages, including two tables. This file also contains information on sampling of participants as well as components of the packets received by participants in *Study 1* and *Study 2*. Page 5 contains the questions participants in *Study 1* answered about injunctive and descriptive civic norms in their neighborhoods. On page 6 are the instructions for the game, followed by examples and test questions on pages 7 through 9. The instructions and test questions were used in *Study 1* and *Study 2*. Participants used pages 10, 11, or 12 to record their responses for the game; these pages appeared in packets for Players 1, 2, and 3, respectively. Page 11 was used by Player 2 in both *Study 1* and *Study 2*. Pages 13 and 14 contain the norms manipulations for *Study 2* participants in Neighborhoods A and B, respectively. Note that in the packet participants were given, we referred to Players A, B, and C rather than Players 1, 2, and 3. In the manuscript, we refer to Players 1, 2, and 3 to avoid confusion with Neighborhoods A and B, which are called such to maintain continuity with Nettle, Colléony & Cockerill (2011).

Study 1 sampling. A total of 562 packets were delivered to Neighborhood A and 819 packets to Neighborhood B. We delivered packets to Neighborhood B at a higher rate because of the lower survey return rate [1] observed in Neighborhood B. We delivered Player 3 packets at a higher rate in both neighborhoods to increase statistical power for modeling *punitiveness*. Variation in the number of successfully delivered packets arose from difficulties in finding the residence or safely accessing the mailslot (particularly in Neighborhood B), as well as changes in residence. As expected, return rates were lower in Neighborhood B (Table S1).

Fourteen participants were excluded from the game because the participant had not completed the game component of the packet. Of these, twelve were from Neighborhood B, and all but one (Player 2) were given the role of Player 3. Because we used the 'strategy method' for Player 3, Player 3s were required to make 10 choices in the game, whereas Players 1 and 2 were required to make only one choice in the game. Most of the potential participants who were excluded for not completing the game circled a choice for only one of the 10 choices required to Player 3. After excluding these 14 participants for incomplete submissions, we have final sample sizes of 40, 44, and 49 for Players 1, 2, and 3, respectively, in Neighborhood A, and 34, 43, and 50 for Players 1, 2, and 3, respectively, in Neighborhood B.

Study 2 sampling. A total of 200 Player 2 packets were delivered to Neighborhood A and 250 packets to Neighborhood B. The return rate for Neighborhood B (16.0%) was within the range observed across roles for *Study 1*. The return rate was slightly lower for Neighborhood A (21.5%) compared to the rate for *Study 1*. We excluded three participants from the game. Of these, two were from Neighborhood A; one participant was excluded for not circling a choice for the game, and the other was excluded because she did not respond to the norms manipulation questions (ESM). We excluded the participant from Neighborhood B because we suspected she had answered the questions randomly (e.g., she indicated

higher trust in people she met for the first time compared than those she knows personally); this did not qualitatively change the results, and we note that she had indicated she expected 3PP. Thus, in *Study 2* we have final samples sizes of 41 and 39 in Neighborhoods A and B, respectively (Table S3).

Table S1. Key sampling values for *Study 1*.

Role	Packets delivered	Response rate	Sample size female	Sample size male
Player 1	163 (241)	24.5% (14.1%)	24 (22)	16 (12)
Player 2	171 (231)	25.7% (19.1%)	22 (20)	22 (23)
Player 3	228 (347)	22.4% (17.6%)	26 (27)	23 (23)

Parentheses contain values for Neighborhood B. Discrepancies between return rates and final sample sizes are due to exclusion of incomplete packets.

Table S2. Key sampling values for *Study 2*.

Role	Packets delivered	Response rate	Sample size female	Sample size male
Player 2	200 (250)	21.5% (16.0%)	20 (23)	21 (16)

Parentheses contain values for Neighborhood B. Discrepancies between return rates and final sample sizes are due to exclusion of incomplete packets.

Here, we ask some questions about certain behaviours. For part 'a' of each question, please tell us whether you think the behaviour is Never OK, Always OK, or somewhere in between. Circle a number from 1 to 10, where 1 is Never OK and 10 is Always OK. Then, for part 'b' of each question, please tell us whether you think No one in your neighbourhood would do this, Everyone in your neighbourhood would do this, or somewhere in between. Circle a number from 1 to 10, where 1 is No one would and 10 is Everyone would.

1. **Cheating the benefits system.**
   a. Do you think it's Never OK, Always OK, or somewhere in between?

1	2	3	4	5	6	7	8	9	10
Never OK									*Always OK*

   b. Do you think many people in your neighbourhood would do this?

1	2	3	4	5	6	7	8	9	10
No one would									*Everyone would*

2. **Avoiding a fare on public transport.**
   a. Do you think it's Never OK, Always OK, or somewhere in between?

1	2	3	4	5	6	7	8	9	10
Never OK									*Always OK*

   b. Do you think many people in your neighbourhood would do this?

1	2	3	4	5	6	7	8	9	10
No one would									*Everyone would*

3. **Cheating on taxes.**
   a. Do you think it's Never OK, Always OK, or somewhere in between?

1	2	3	4	5	6	7	8	9	10
Never OK									*Always OK*

   b. Do you think many people in your neighbourhood would do this?

1	2	3	4	5	6	7	8	9	10
No one would									*Everyone would*

Now we explain the game to you. You will play the game with other people in your neighbourhood.

About the Game

You can get real money from the game. Any money you make will be delivered to you in cash, in a sealed envelope, along with the £5 thank-you money. We will not keep any information linking your name or address to the choices you made in the game. We assure you the money is real, will be delivered in cash within one week from when we get your packet, and carries no conditions.

The Game

You will play this game with 2 other people who also got this packet in the post. These people are from your neighbourhood. You will never know who they are. They will never know who you are either. Each player is given a role in the game. The roles are: Player A, Player B, and Player C. The role you will play is given by the packet you got. The same is true for the other players in the game.

How the game is played in the post:

We provide an initial amount of £10 to **each** Player. Players A and C each make a choice about what to do with their money. They write this choice in their packets. The game is played once the packets have been returned to the university by post. Then, we deliver in cash the money each of the players got from the game. With the cash, we give each player a sheet showing what the other players chose to do.

This is what happens in the game:

Players A, B, and C **each** get £10 to start the game with. Player A must decide whether to take none, some, or all of the £10 that Player B would have gotten. If Player A takes money from Player B, then Player A will have it for themselves and Player B will not have it. Then, Player C must decide whether to use some of their money to make Player A lose money.

For each possible amount Player A might take from Player B (from £1 to £10), Player C makes a decision. Player C must decide whether to pay £2 to reduce the money Player A gets by £6 **or** to “do nothing”. If Player C decides to do nothing, they keep their full £10.

Note:

If Player A chooses to take £0 from Player B (that is, they choose not to take money from Player B), Player C cannot pay to make Player A lose money. Also, whatever Player C chooses, Player B will not get back the money Player A took from them if Player A chose to do so.

Because Player B cannot make a choice in the game, we ask Player B what they think Player C will do.

How the Game Works

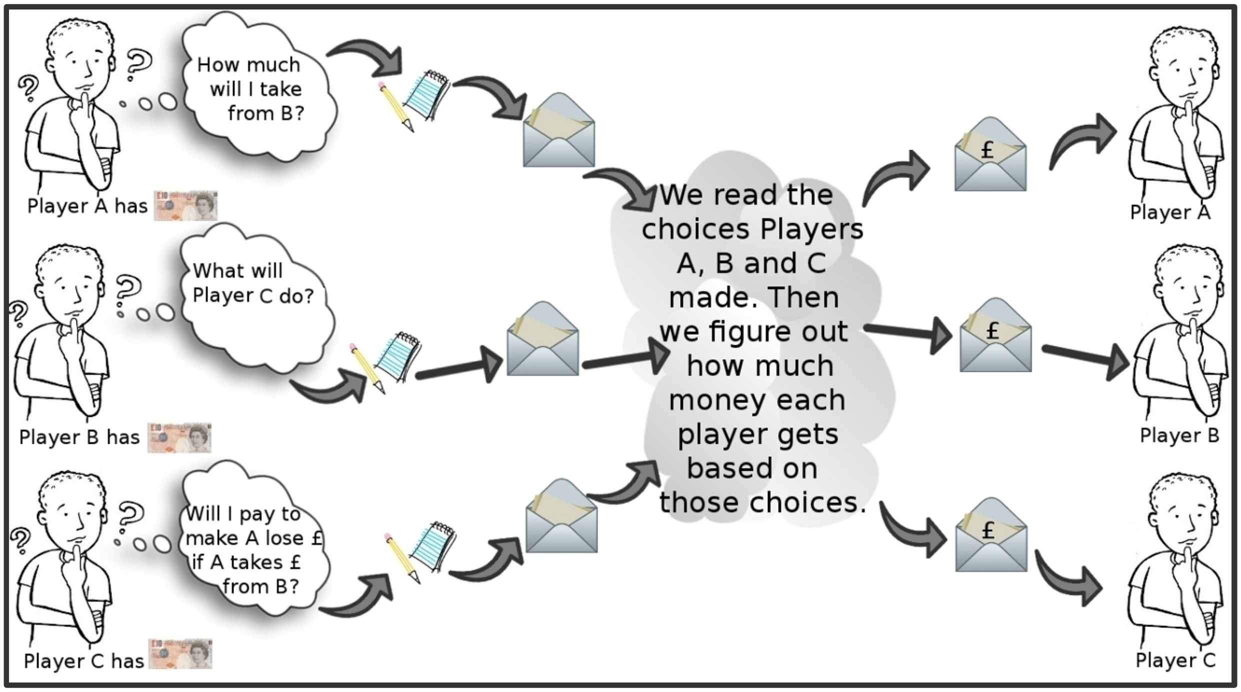

On this page and the next 3 pages, we go over examples. In Examples 3-5, we ask you questions to show you understand the game. The questions are in boxes. ***Make sure you answer all the questions before playing the game for real!*** *Then we tell you which role you will play and you will play for real.*

Example 1.

Players A, B, and C each start with £10.

Player A ***Player B*** ***Player C***

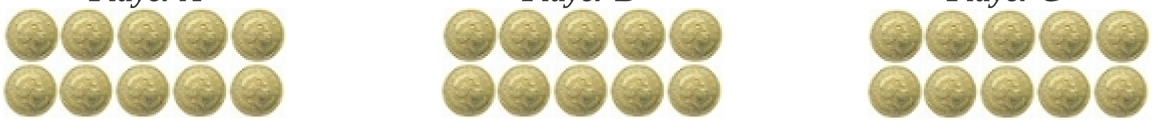

Suppose Player A decided to take £0 from Player B.

In this case, Player A still has £10. Player B still has £10 as well. And Player C cannot pay to make Player A lose money, so Player C also still has £10.

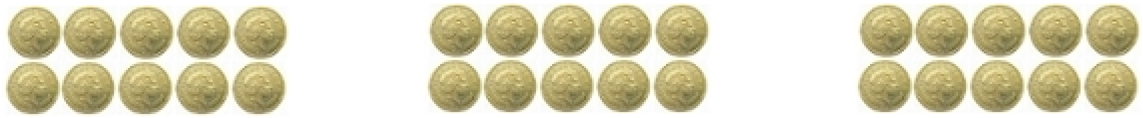

Player A ***Player B*** ***Player C***

<u>Example 2.</u>

Players A, B, and C each start with £10.

Player A ***Player B*** ***Player C***

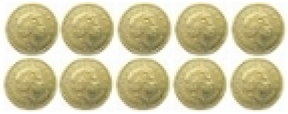 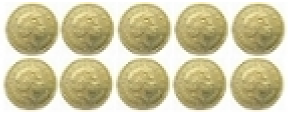 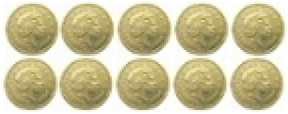

Suppose Player A decided to take £1 from Player B. Now Player A has £11 and Player B has £9.

Player A ***Player B*** ***Player C***

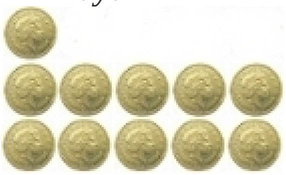 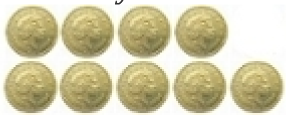 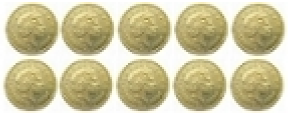

Now, suppose Player C had decided to "do nothing" if Player A were to take £1 from Player B. Then, Player A still gets £11. Player B still gets £9. And Player C still gets £10.

Player A ***Player B*** ***Player C***

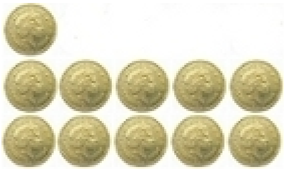 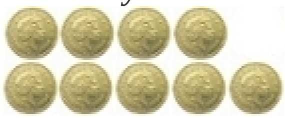 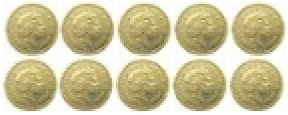

<u>Example 3.</u>

Players A, B, and C each start with £10.

Player A ***Player B*** ***Player C***

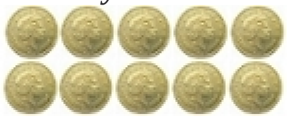 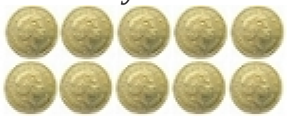 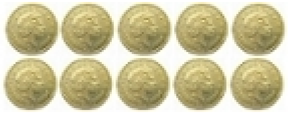

Suppose Player A decided to take £10 from Player B. Now Player A has £20.

<u>QUESTION</u>: How many £ does Player B have now (fill in the blank)? **£____**

Player A ***Player B*** ***Player C***

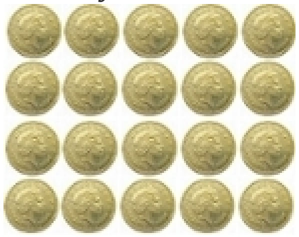

?

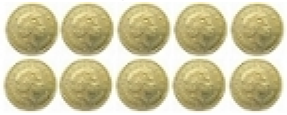

However, suppose Player C had decided that if Player A were to take £10 from Player B, they would pay to make Player A lose money.

Then, Player C pays £2 to subtract £6 from the money Player A was going to get. Player C would then get £8 (£10 minus £2 equals £8). Player B would still get £0.

<u>QUESTION</u>: How many £ does Player A get? **£____**

Player A Player B Player C

? **0**

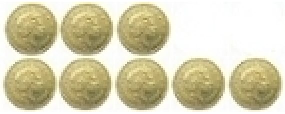

Example 4.

Players A, B, and C each start with £10.

Player A	***Player B***	***Player C***
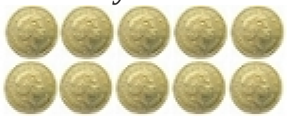	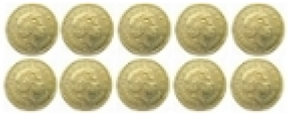	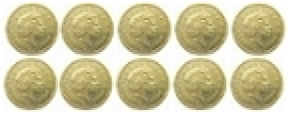

Suppose Player A decided to take £1 from Player B. Now Player A has £11 and Player B has £9.

Player A	***Player B***	***Player C***
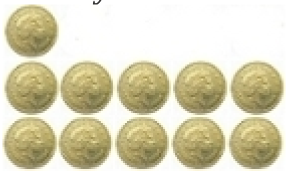	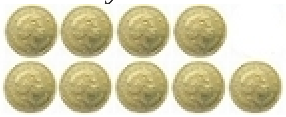	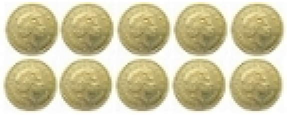

However, suppose Player C had decided that if Player A were to take £1 from Player B, they would pay to make Player A lose money.

Then, Player C pays £2 to subtract £6 from the money Player A was going to get. Player A would get £5 (£11 minus £6 equals £5). Player B would still get £9.

QUESTION: How many £ would Player C get? **£____**

Player A	***Player B***	***Player C***
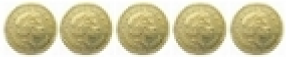	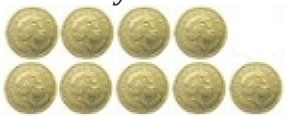	**?**

Example 5.

Players A, B, and C each start with £10.

Player A	***Player B***	***Player C***
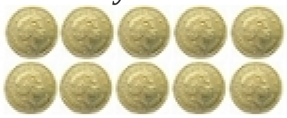	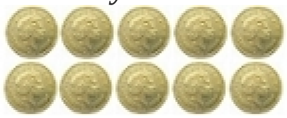	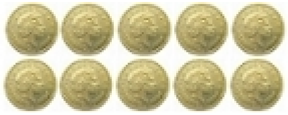

Suppose Player A decided to take £10 from Player B. Now Player A has £20 and Player B has £0.

Player A	***Player B***	***Player C***
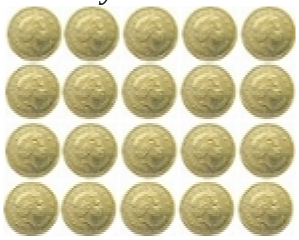	**0**	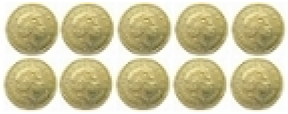

Now, suppose Player C had decided to “do nothing” if Player A were to take £10 from Player B.

QUESTIONS: How many £ would Player A get? **£____** Player B? **£____** Player C? **£___**

Player A	***Player B***	***Player C***
**?**	**?**	**?**

We have gone over examples of the game. ***Be sure you filled in answers to all the questions in boxes that tested your understanding of the game!*** *On this page, you will* ***NOW*** *play the game* **for real**.

<u>THE GAME</u>

Your role is: **Player A**.

You are starting the game with £10. Players B and C are starting the game with £10 as well. Along with any money from the game, each player will receive a sheet that explains what decisions were made in the game and the outcomes.

How many pounds do you choose to take from Player B?

<u>Instructions:</u> **Circle** an amount.

£0

£1

£2

£4

£5

£6

£7

£8

£9

£10

Check – Be sure you circled your choice! To play the game, you need to make a decision!

We have gone over examples of the game. ***Be sure you filled in answers to all the questions in boxes that tested your understanding of the game!*** *On this page, you will* **NOW** *play the game* **for real**.

THE GAME

Your role is: **Player B**.

You are starting the game with £10. Players A and C are starting the game with £10 as well. Along with any money from the game, each player will receive a sheet that explains what decisions were made in the game and the outcomes.

You cannot make a choice in this game. The amount of money you make in the game will depend on what Player A does. **However**, **we would like to know what you think Player C would choose to do.** Please fill out Question 1.

Question 1. Suppose Player A decided to take **£5** from Player B (you). If Player A took £5 from you, do you think that Player C would choose to pay £2 of their £10 to make Player A get £6 less?

Instructions: **Circle** the choice you **think** Player C would make **if Player A took £5 from you**.

Do nothing **OR** Pay £2 so Player A gets £6 less

We have gone over examples of the game. ***Be sure you filled in answers to all the questions in boxes that tested your understanding of the game!*** *On this page, you will* ***NOW*** *play the game* ***for real****.*

<u>THE GAME</u>

Your role is: **Player C**.

You are starting the game with £10. Players A and B are starting the game with £10 as well. Along with any money from the game, each player will receive a sheet that explains what decisions were made in the game and the outcomes.

We will not know how many pounds Player A will take from Player B until the packets are received at the university. So, you must decide ahead of time what you choose to do for each decision Player A could make.

<u>Instructions:</u> For **EACH possible amount Player A could take** from Player B, **circle one of the options**. There are **10 choices for you to make**.
Note: If Player A does not take any money from Player B, you cannot pay to reduced the amount of money Player A gets. So, there is no choice for you to make if Player A takes £0.

	**IF . .**	**I CHOOSE TO . .**	
**Choice 1.**	Player A takes **£1** from Player B	Do nothing	Pay £2<br>so Player A loses £6
**Choice 2.**	Player A takes **£2** from Player B	Do nothing	Pay £2<br>so Player A loses £6
**Choice 3.**	Player A takes **£3** from Player B	Do nothing	Pay £2<br>so Player A loses £6
**Choice 4.**	Player A takes **£4** from Player B	Do nothing	Pay £2<br>so Player A loses £6
**Choice 5.**	Player A takes **£5** from Player B	Do nothing	Pay £2<br>so Player A loses £6
**Choice 6.**	Player A takes **£6** from Player B	Do nothing	Pay £2<br>so Player A loses £6
**Choice 7.**	Player A takes **£7** from Player B	Do nothing	Pay £2<br>so Player A loses £6
**Choice 8.**	Player A takes **£8** from Player B	Do nothing	Pay £2<br>so Player A loses £6
**Choice 9.**	Player A takes **£9** from Player B	Do nothing	Pay £2<br>so Player A loses £6
**Choice 10.**	Player A takes **£10** from Player B	Do nothing	Pay £2<br>so Player A loses £6

<u>Check – Be sure you circled an option for each of the 10 choices! To play the game, you need to make a decision for each of the 10 choices!</u>

As a part of the Tyneside Neighbourhoods Project, we recently asked 10 people in your neighbourhood how common they think certain behaviours are in your neighbourhood. For each behaviour, we asked them to circle a number from 1 to 10, where 1 is No one in your neighbourhood would do this and 10 is Everyone in your neighbourhood would do this.

We then averaged their answers to get an idea of how common people think certain behaviors are in your neighbourhood. Below we show you what they think. What do you think of their answers?

1. We asked: <u>Would many people in your neighbourhood cheat the benefits system</u>?

People in your neighbourhood think:

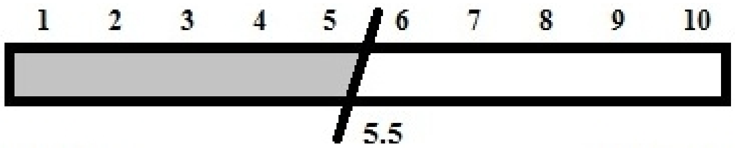

No one would *Everyone would*

What do you think? (circle one)

Fewer people would do this This is about right More people would do this

2. We asked: <u>Would many people in your neighbourhood avoid a fare on public transport</u>?

People in your neighbourhood think:

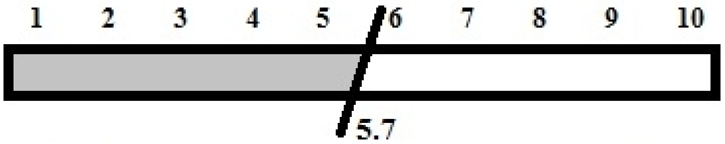

No one would *Everyone would*

What do you think? (circle one)

Fewer people would do this This is about right More people would do this

3. We asked: <u>Would many people in your neighbourhood cheat on taxes</u>?

People in your neighbourhood think:

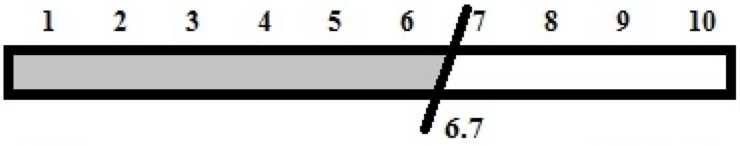

No one would *Everyone would*

What do you think? (circle one)

Fewer people would do this This is about right More people would do this

As a part of the Tyneside Neighbourhoods Project, we recently asked 10 people in your neighbourhood how common they think certain behaviours are in your neighbourhood. For each behaviour, we asked them to circle a number from 1 to 10, where 1 is No one in your neighbourhood would do this and 10 is Everyone in your neighbourhood would do this.

We then averaged their answers to get an idea of how common people think certain behaviors are in your neighbourhood. Below we show you what they think. What do you think of their answers?

1. We asked: <u>Would many people in your neighbourhood cheat the benefits system</u>?

People in your neighbourhood think:

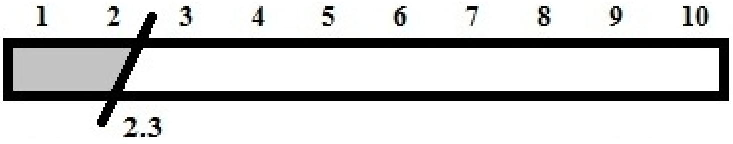

No one would *Everyone would*

What do you think? (circle one)

Fewer people would do this This is about right More people would do this

2. We asked: <u>Would many people in your neighbourhood avoid a fare on public transport</u>?

People in your neighbourhood think:

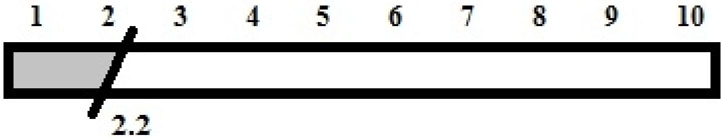

No one would *Everyone would*

What do you think? (circle one)

Fewer people would do this This is about right More people would do this

3. We asked: <u>Would many people in your neighbourhood cheat on taxes</u>?

People in your neighbourhood think:

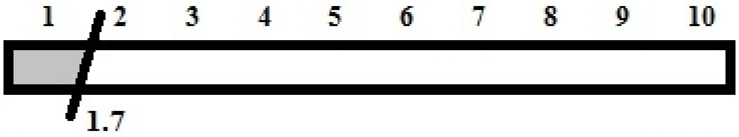

No one would *Everyone would*

What do you think? (circle one)

Fewer people would do this This is about right More people would do this

References.

Nettle D, Colléony A, Cockerill M. 2011 Variation in cooperative behaviour within a single city. *PloS One* **6**:e26922. doi:10.1371/journal.pone.0026922.
